# qNeck Trauma and Extra-tracheal Intubation

**Published:** 2018-02-28

**Authors:** Vinh K. Pham, Justin C. Sandall

**Affiliations:** Department of Anesthesiology, University of Kansas School of Medicine-Wichita

**Keywords:** wounds and injuries, neck injuries, airway management, intubation

## Introduction

Neck trauma can be devastating due to the relatively high probability of life-threatening pathology. Although direct neck injury is uncommon and only accounts for 1:30,000 emergency room visits,^1^ airway trauma remains a major cause of early death in trauma.^2^,^3^ Airway obstruction or associated injury (i.e., retropharyngeal hematoma) is estimated to account for a 30% early mortality rate.^2^,^4^ Early recognition and security of the airway remains a priority in resuscitating patients with airway compromise. However, there is limited evidence on how to manage traumatic airways. This is likely due to the unpredictable nature of the injuries themselves and the rarity of these events.

## Case Presentation

A 21-year-old male was brought to the emergency room as a level one trauma patient after suffering a suspected brain injury in a forklift accident. Upon arrival, emergency medical services remarked that the patient was initially pulseless and apneic, thus cardiopulmonary resuscitation had been initiated. His cervical spine was secured with a C-collar, and a Combitube™ placed to facilitate ventilation. Return of spontaneous circulation (ROSC) was achieved after ten minutes and two doses of epinephrine.

**Figure 1:**
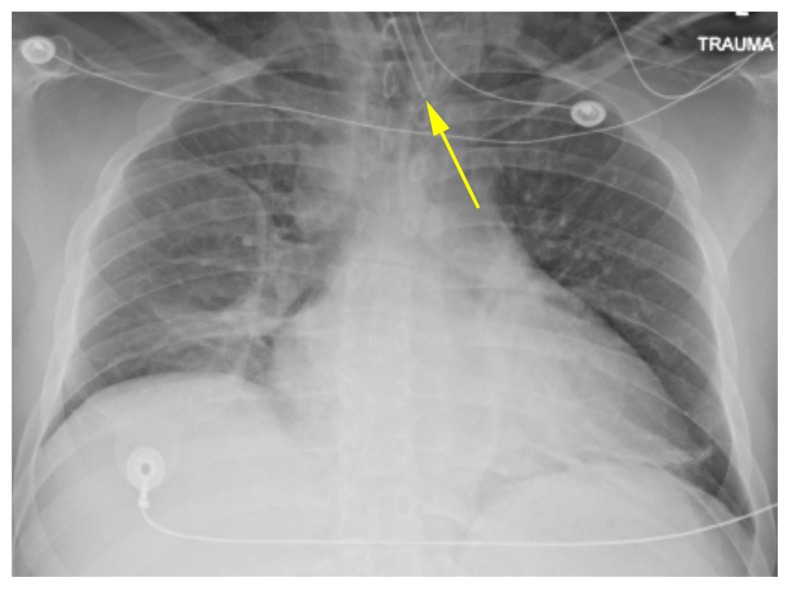
Chest x-ray depicts the ET tube placement.

The patient had a Glasgow Coma Score of 3 and needed a more secure airway to facilitate imaging and possible operative intervention. He was oxygenated with 100% FiO2, and the Combitube™ was removed under visualization utilizing the Glidescope_®_. The airway was assessed while maintaining in-line cervical stabilization. Blood was seen throughout the oropharynx and through the glottic aperture. An 8.0 endotracheal (ET) tube was placed under visualization; placement was confirmed with positive end-tidal CO_2_ of 75. SaO_2_ reached 88% at the lowest point, but promptly improved with recruitment maneuvers. The patient was hyperventilated in an attempt to normalize CO_2_ and attenuate cerebral vasodilation that could lead a potential increase in intracranical pressure. Figure 1 depicts a chest x-ray showing the ET tube projecting over the trachea, but the tip appeared to be lateral to the tracheal lumen.

**Figure 2:**
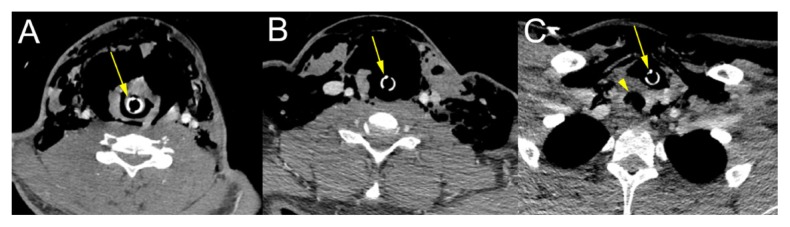
Transverse images of a CT of the neck show path of the ET tube (arrow): A) CT scan showing ET tube passing through glottis; B) ET tube moving inferior, anterior airway noted to have possible disruption; and C) ET tube is noted to be outside of the trachea (arrowhead).

**Figure 3:**
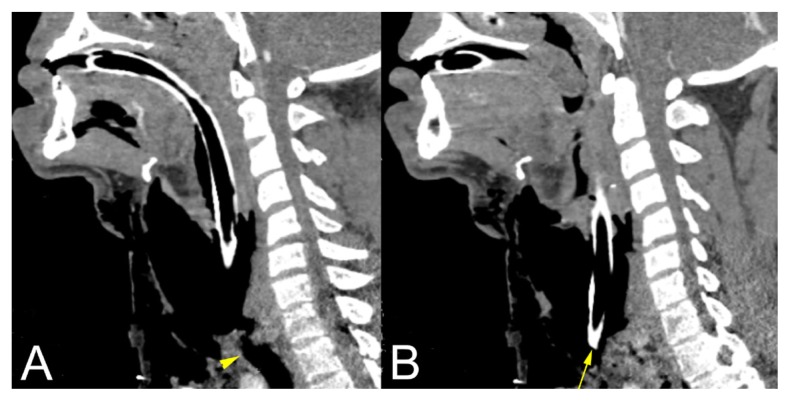
Sagittal images of a CT of the neck show the ET tube location relative to trachea: A) note the location of the trachea and trajectory of the ET tube, B) the ET tube tip lies anterior to the trachea.

A computed tomography (CT) scan of the head showed no acute intracranial abnormality, but demonstrated extensive soft tissue gas throughout the neck bilaterally extending into the right face. A CT angiogram of the neck revealed multiple nondisplaced transverse process fractures and an apparent lack of vertebral artery flow bilaterally. A vascular surgery consultant suspected bilateral vertebral artery dissection with occlusion. A CT of the neck and chest (Figures 2 and 3) showed extensive pneumomediastinum, gas throughout chest, neck, and face, and, most notably, the ET tube was anterior to the trachea.

The anesthesiology team learned of the mechanism of his injury at this time. He had leaned forward under the forklift crossbar, his knee hit a lever on his control board, lowering the forklift, and his neck was caught between the crossbar and a fixed strut. Witnesses reported his body lay on the lever, and they were unable to raise the lift off his neck for five minutes. Large contusions were noted on the anterior and posterior neck with significant subcutaneous emphysema.

The patient was transported emergently to the operating room to undergo a bronchoscopy through the endotracheal tube (ETT) to visualize the tracheal injury and reposition the ETT. The patient’s vital signs remained stable, including O_2_ saturation remaining above 95%. Qualitative end-tidal CO_2_ was positive. The patient was at high risk for loss of the airway, thus the surgical team was present during the entire procedure to provide a surgical one, if necessary. Bronchoscopy noted friable and erythematous tissue at the distal end of the ETT, and unknown particulate matter also was seen. The ETT was withdrawn until a slit area was noted in the right visual field; the bronchoscope was advanced into this opening. Tracheal rings were appreciated and the ETT was advanced toward the carina. A probable linear tear was visualized in the anterior trachea/larynx as the ETT was withdrawn. A Glidescope_®_ was inserted to enable visualization of the ETT superior to the glottis. The bronchoscope was reinserted anterior to the ETT, and bubbles of blood were noted distal to the glottis coinciding with an area of suspected tracheal perforation. Initial ETT cuff inflation likely served to tamponade local bleeding. The tracheal discontinuity could not be well-visualized due to the surrounding hemorrhage. Ventilation improved, oxygenation remained stable, and there was no hemodynamic collapse from known pneumomediastinum.

A follow-up CT of the neck was performed to re-assess the patient’s cervical spine. It also showed continued tracheal placement of the ETT. Unfortunately, the patient sustained a severe anoxic brain injury. An electroencephalogram showed severe diffuse encephalopathy and findings consistent with coma. A magnetic resonance image (MRI) of the brain, 48 hours post-trauma, showed findings consistent with global hypoxic ischemic injury without evidence of herniation, hydrocephalus, or midline shift. An MRI of the cervical spine showed a transected cervical spinal cord injury. No improvements in neurological status were observed. His family pursued comfort care measures and organ donation according to the patient’s wishes.

## Discussion

Injuries to the airway are rare and account for less than 1% of emergency room visits.^3^ However, there remains limited evidence on how to manage a traumatized airway, and effectively securing it can be fraught with unforeseen challenges. This is likely due to the unpredictable nature of the injuries themselves and the rarity of these events. Early recognition and security of the traumatic airway remain vital in resuscitating patients.

Head and neck injuries can be complex, and typically, the neck can be divided into three zones to help with a differential diagnosis.^2^,^5^ Zone I is below the cricoid and has a relatively significant mortality rate of 12% due to high likelihood of injury to the great vessels. Angiography usually is recommended over manual exploration if the patient is stable. Zone II lies between the cricoid and mandible and consists of 60 – 75% of neck injuries. Zone III is the area superior to the mandible, primarily consisting of bony facial structures at risk of fracture and displacement.

Recognizing laryngeal injury early is vital in ensuring the proper treatment to sustain both life and phonation. In general, neck trauma is subcategorized into blunt and penetrating trauma. Laryngeal-tracheal separation can occur with both types and even following endotracheal tube placement.^6^–^8^ A completely transected cervical trachea has the risk of retracting into the mediastinum, likely necessitating immediate surgical intervention.^2^ In addition, after airway manipulation, a delayed tracheobronchial disruption may prove difficult to recognize without high suspicion and even more challenging to treat.^1^ In our case, recognition of airway injury was delayed due to initial lack of information regarding mechanism of injury as well as the presence of a cervical collar that obscured evidence of neck trauma. In retrospect, bronchoscopy immediately following intubation could have been considered.

Neck injuries introduce a myriad of complications to airway security, including hemorrhage, aspiration, retropharyngeal hematoma,^9^ skull base fracture, and temporomandibular joint injury limiting mouth opening. Cervicothoracic vascular injuries are reported to occur in up to 25% of neck trauma.^4^ Cervical spine injury at C4-5 increases the risk of apnea secondary to loss of diaphragmatic innervation and neurogenic shock.^2^,^10^ Only a few other cases of pneumopericardium or pneumoperitoneum from hypopharyngeal perforation have been reported previously, which can increase the risk of cardiovascular collapse. Due to its infrequency, however, this risk may not warrant an extensive workup.^11^–^13^ Injury to the esophagus is more common in penetrating trauma than blunt trauma.^4^

A current proposed airway strategy for trauma cases include: 1) the larynx and trachea must be clearly intact and in continuity versus partial separation or avulsions of these structures, 2) the airway should be visible to inspection by endoscopy in the emergency department or operating room, and 3) intubation requires a highly experienced physician.^14^,^15^ If available, fiberoptic laryngoscopy should be utilized to assess the airway patency, vocal fold mobility, and integrity of the pharynx and larynx.^14^ Use of video laryngoscopy or lighted stylet reduce cervical neck motion when compared with direct laryngoscopy with a Macintosh blade and should be considered in trauma cases.^16^–^18^ If a definitive airway is unable to be established, an emergency cricothyroidotomy or surgical tracheostomy is warranted.^2^

After securing the airway, ongoing debate continues for obligatory exploration versus selective exploration, some arguing for a more conservative approach with the latter.^11^ Ongoing case reports and series will build a foundation of literature pertaining to airway trauma. In time, sufficient information may be gathered to form a consensus with how to best approach these uncommon presentations to maximize the likelihood of meaningful survival.
